# Ratiometric photoacoustic imaging of endoplasmic reticulum polarity in injured liver tissues of diabetic mice†
†Electronic supplementary information (ESI) available. See DOI: 10.1039/c7sc02330h



**DOI:** 10.1039/c7sc02330h

**Published:** 2017-08-14

**Authors:** Haibin Xiao, Chuanchen Wu, Ping Li, Wen Gao, Wen Zhang, Wei Zhang, Lili Tong, Bo Tang

**Affiliations:** a College of Chemistry, Chemical Engineering and Materials Science , Institute of Biomedical Sciences , Collaborative Innovation Center of Functionalized Probes for Chemical Imaging in Universities of Shandong , Key Laboratory of Molecular and Nano Probes , Ministry of Education , Shandong Normal University , Jinan 250014 , PR China . Email: tangb@sdnu.edu.cn ; Email: lip@sdnu.edu.cn

## Abstract

As one of the complications of diabetes, liver injury results in significant hazards. Therefore, accurately diagnosing diabetes-induced liver injury beforehand is crucial for the warning and treatment of hepatic diseases. Diabetes-induced liver injury can cause changes in the microstructure and morphology of liver tissue, leading to changes in the hydrophilic and hydrophobic domains in the endoplasmic reticulum (ER), which is closely associated with changes in cellular ER polarity. So, differences in the ER polarity can indicate the degree of diabetes-induced liver injury. Herein, we develop a new fluorescent and photoacoustic dual-mode probe, ER-P, for detection of the ER polarity of liver tissue in normal and diabetic mice. Upon excitation with a 633 nm laser, ER-P showed increasing fluorescence intensity at 800 nm accompanying a decline in the polarity. Due to its polarity-sensitivity, ER-P was utilized for confocal fluorescence imaging in live cells, and the results demonstrate that ER-P can exclusively accumulate in the ER and indicate an increase in the polarity during ER stress. Importantly, ER-P displayed different absorbance intensities at 700 nm and 800 nm in different polarity environments because of intramolecular charge transfer. The photoacoustic intensity ratios between 700 nm and 800 nm will enable quantification of polarity to be achieved. The ratiometric photoacoustic imaging data demonstrate that the polarity of the liver tissue of diabetic mice is higher than that of the liver tissue of normal mice. Meanwhile, after treatment with the antidiabetic drug metformin, diabetic mice exhibit a reduced polarity environment in their liver tissue. The proposed study may serve as a new approach for the early diagnosis and therapeutic evaluation of diabetes-induced liver injury.

## Abstract 2

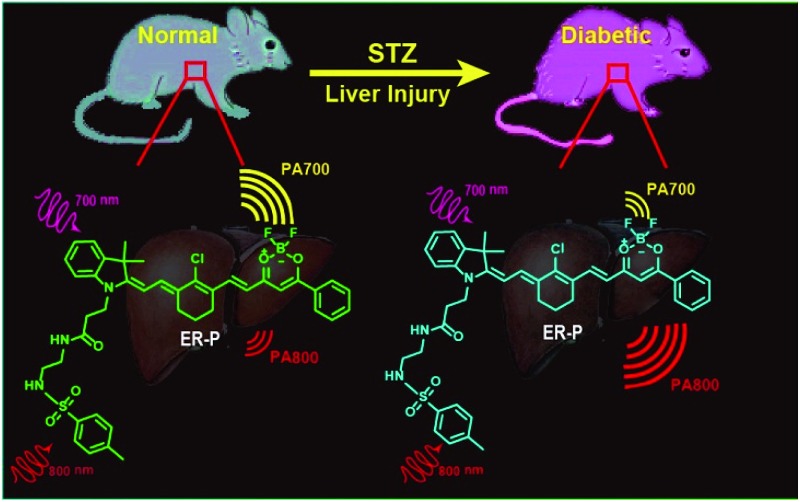
We have developed a new fluorescent and photoacoustic dual-mode probe, ER-P, for the detection of ER polarity of liver tissues in normal and diabetic mice.

## Introduction

Diabetes mellitus is a metabolic disease that results in significant morbidity and mortality.^1^ In particular, as one of the complications of diabetes, liver injury is a major problem occurring in diabetic patients.^2–6^ Diabetes-induced liver injury can cause necrosis of hepatocytes, inflammation and fibrosis of liver tissue and development of nonalcoholic fatty liver disease, seriously threatening human health.^7,8^ Hence, early and accurate diagnosis of diabetes-induced liver injury is essential for the warning and treatment of hepatic diseases. To date, the diagnostic techniques for diabetes-induced liver injury mostly focus on isolated staining of tissue sections and the determination of related markers with different kits.^9,10^ Obviously, these methods are tedious and deviate from the true living tissue, which presents obstacles for the precise treatment of diabetes complications. Therefore, it is imperative to develop an *in situ*, nondestructive, instantaneous method for detecting and determining the degree of diabetes-induced liver injury.

An increasing number of studies have reported that diabetes-induced liver damage is evident from many changes in the microstructure and morphology of liver tissue, such as vacuolization. In addition, liver tissue from diabetic rats shows a significant increase in the levels of reactive oxygen species (ROS), aspartyl aminotransferase, alkaline phosphatase, alanine aminotransferase and bilirubin, meanwhile a dramatic decrease in the levels of glutathione (GSH), glutathione-S-transferase, quinone reductase, catalase, and superoxide dismutase.^11–14^ The variation in the content of these proteins or enzymes could accompany the changes in the hydrophilic and hydrophobic domains in the endoplasmic reticulum (ER), which are closely related to ER stress and polarity changes. Therefore, it is speculated that compared with normal liver tissue, the liver tissue of diabetic patients appears to have a distinguished polarity within the ER.^15,16^ This difference in polarity can indicate the degree of diabetes-induced liver injury. However, to the best of our knowledge, there is no appropriate tool that can specifically monitor the subtle changes in ER polarity to illustrate liver injury.

Photoacoustic (PA) imaging is an excellent emerging *in vivo* imaging modality.^17,18^ It depends on the translation of excitation light into ultrasonic waves based on the PA effect, thus providing deeper tissue penetration and higher *in vivo* spatial resolution.^19,20^ Therefore PA imaging has been widely used in biology and medicine as a noninvasive imaging approach.^21,22^ Recently, PA imaging methods have been broadly applied to tumor imaging^23–39^ and visualization of various active molecules, such as peroxynitrite, hydrogen peroxide, hydrogen sulfide, metal ions, aminothiol and so on.^40–46^ However, up until now a PA imaging probe that can quantify polarity, especially ER polarity, has not been presented. In view of this, there is an urgent need to construct a superior PA imaging probe for the detection of polarity in deep tissue, realizing the diagnosis of polarity-related diseases such as diabetes-induced liver injury.

**Scheme 1:**
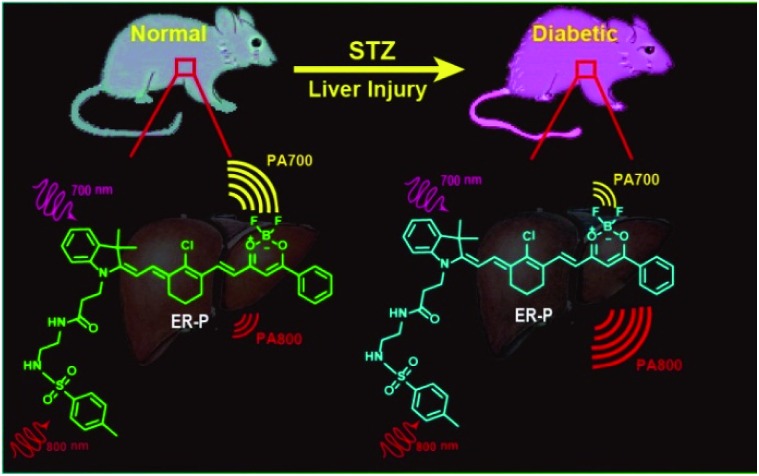
The structure of the photoacoustic probe ER-P and the corresponding mechanism.

To achieve the aforementioned goal, we fabricated a new near-infrared (NIR) fluorescent and ratiometric PA imaging dual-mode probe termed ER-P for visualization of the polarity within the liver tissue of diabetic mice (Scheme 1). Structurally, ER-P comprises a merocyanine moiety containing a tertiary amine as an electron donor and a difluoroboronate moiety as an electron acceptor. The long conjugated system of the probe produces the NIR absorption spectra, endowing it with the ability to generate an intense PA signal. Due to intramolecular charge transfer (ICT), the maximum absorption wavelength of ER-P obviously red-shifts with an increase in the environmental polarity, resulting in changes in the PA signal intensities at two selected wavelengths – 700 nm (PA700) and 800 nm (PA800). The PA signal intensity ratios between these two wavelengths (PA700/PA800) can quantify the polarity of the media. In addition, the fluorescence intensity of ER-P at 800 nm decreases dramatically with increasing polarity of the media, which can enable fluorescence detection of the polarity. A methyl sulphonamide moiety was introduced into ER-P to assist the probe in accumulating in the ER by binding to the sulphonamide receptor.^47^ The present experimental data indicated that ER-P could sensitively and selectively respond to the polarity of the environment. By utilizing confocal fluorescence imaging, we verified that ER-P could solely permeate into the ER. Moreover, the polarity variations in the ER upon different stimuli were monitored using ER-P. More importantly, by utilizing PA imaging, the polarity differences of liver tissue in normal and diabetic mice were visualized *in vivo* for the first time.

## Results and discussion

### Synthesis of the probe ER-P

The synthesis of ER-P is outlined in Scheme S1 of the ESI.† First, the ER-targetable indolium iodide 3 was obtained by reacting 1-(2-carboxyethyl)-2,3,3-trimethyl-3*H*-indolium iodide 1 with *N*-tosyl-ethylenediamine 2 through an amidation reaction. Then a facile one-pot reaction of 2-chloro-1-formyl-3-hydroxymethylenecyclohexene 4, 5 and 3 in acetic anhydride obtained the final compound ER-P. All of the synthesis conditions and yields were moderate. The structure of ER-P was well characterized by ^1^H NMR, ^13^C NMR, and HR-MS.

### *In vitro* fluorescence and PA responses to solvents with different dielectric constants

**Fig. 1:**
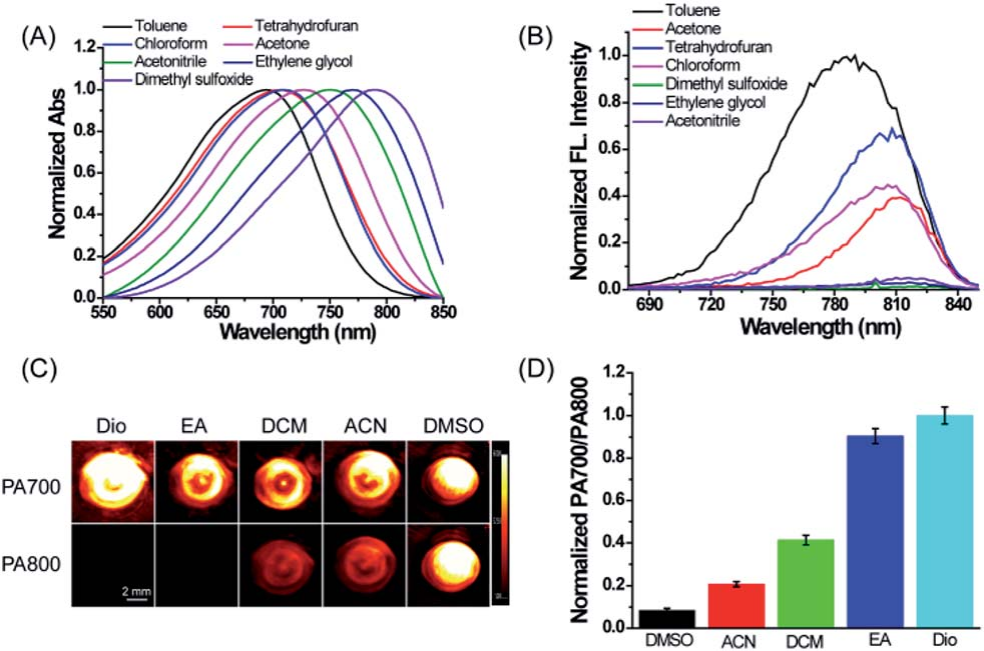
The spectroscopic and PA responses of ER-P in various solvents with different polarity indices. (A and B) The absorption and fluorescence spectra of ER-P (10 μM, 1% DMSO as a cosolvent) in seven solvents with different polarities. (C and D) Representative PA images and PA intensity ratios of ER-P (50 μM, 1% DMSO as a cosolvent) in five solvents with different polarities. Dio, dioxane; EA, ethyl acetate; DCM, dichloromethane; ACN, acetonitrile; DMSO, dimethyl sulfoxide.

First, we evaluated the spectroscopic properties and PA signal of ER-P in common solvents with different dielectric constants and polarity indices in detail. As shown in Fig. 1A, the maximum absorption wavelengths of ER-P red-shifted obviously with an increase in the solvent polarity index. For example, they ranged from about 694 nm in toluene (dielectric constant *ε* = 2.37 and polarity index *P* ≈ 2.3) to about 794 nm in dimethyl sulfoxide (DMSO, *ε* = 47.2 and polarity index *P* ≈ 7.2). Meanwhile, the fluorescence emission spectra of ER-P exhibited obvious polarity-dependence upon excitation at 633 nm (Fig. 1B). The probe showed intense fluorescence and higher quantum yields in lower polarity index media. By contrast, it displayed weaker fluorescence and lower quantum yields in higher polarity index media. For example, the fluorescence intensity of ER-P at 800 nm in toluene was about 70-fold higher than that in DMSO. The detailed spectroscopic properties of ER-P are summarized in Table S1.† Furthermore, due to the obvious red shift of the absorbance maxima accompanying the increase in the dielectric constant of the media, ER-P will produce different PA signals at two selected wavelengths (700 nm and 800 nm), from which the PA intensity ratios (PA700/PA800) can be determined. In lower polarity media, the absorbance intensity at 700 nm is larger than that at 800 nm, resulting in a larger PA700/PA800 ratio. Conversely, in higher polarity media, ER-P shows an increase in the absorption intensity at 800 nm with an accompanying decrease in the absorption intensity at 700 nm, which will cause a small PA700/PA800 ratio. To verify this assumption, we tested the PA signals of ER-P at two excitation wavelengths, 700 nm and 800 nm, in five representative solvents with different dielectric constants and polarity indices. As illustrated in Fig. 1C, following the increase in the polarity from dioxane (Dio) to DMSO, the PA signal of ER-P at 700 nm (PA700) reduced gradually, in contrast to the PA signal of ER-P at 800 nm (PA800), which enhanced rapidly. By quantifying the PA signal intensity the PA intensity ratios of PA700/PA800 were obtained and are shown in Fig. 1D, displaying a higher ratio in lower polarity. All of these results demonstrate that ER-P is an ideal probe for both fluorescence and PA responses to polarity within different polarity environments.

### *In vitro* quantification of the dielectric constants of water–dioxane mixtures

**Fig. 2:**
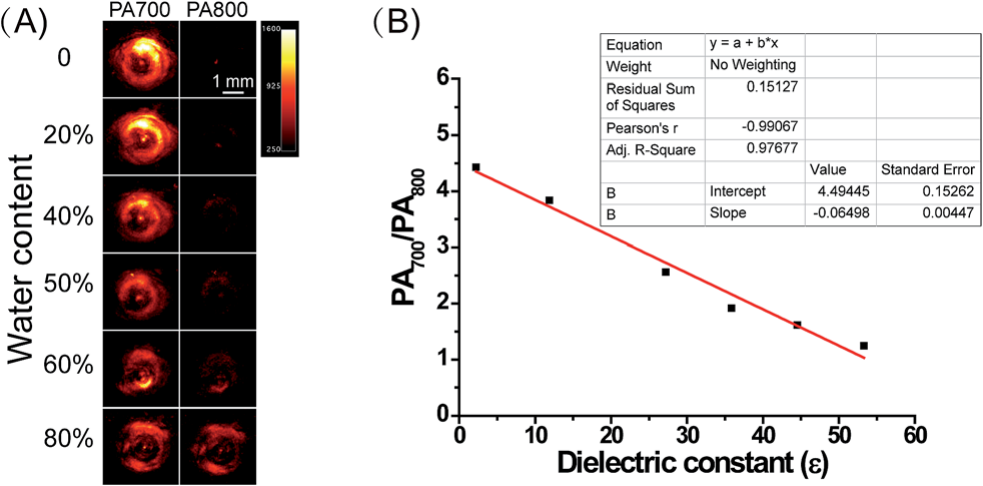
(A) Representative PA images of ER-P (10 μM, 1% DMSO as a cosolvent) with excitation at 700 and 800 nm in dioxane–water mixtures (water from 0 to 80%). (B) The linear relationship between the PA intensity ratios of ER-P (10 μM) and dielectric constant.

Subsequently, we attempted to quantify the precise polarity of certain media with ER-P through the PA intensity ratios PA700/PA800. The PA intensity ratios PA700/PA800 of ER-P were investigated in mixtures of dioxane and water. The mixtures with increasing proportions of water represented an increase in polarity. As outlined in Fig. 2A, when the dielectric constant increased with the increase in water content in the water–dioxane mixture, the PA signal intensity of ER-P showed a dramatic decrease at 700 nm and increase at 800 nm. There was good linearity between the PA intensity ratios PA700/PA800 and the dielectric constants of the mixtures (Fig. 2B), and the correlation equation is *y* = –0.06498*x* + 4.49445. Therefore, ER-P can be used to quantify the environmental polarity by detection of its PA intensity ratios PA700/PA800. Additionally, the fluorescence emission spectra of ER-P exhibited remarkable polarity dependence in the water–dioxane mixtures. The fluorescence intensity decreased significantly with the increase in the dielectric constant (Fig. S1†). Together these data verified that ER-P is a polarity-sensitive fluorescence and PA dual mode probe.

### The fluorescence and PA selectivity of ER-P

**Fig. 3:**
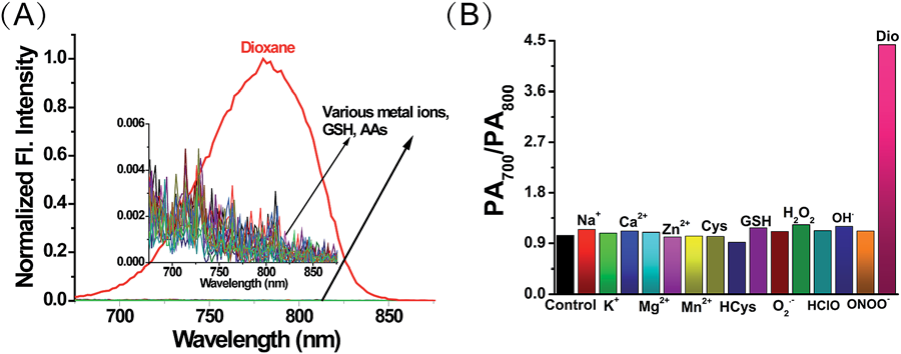
(A) The fluorescence spectra of ER-P (10 μM, 1% DMSO as a cosolvent) in the presence of various interferents. (B) The PA intensity ratios of ER-P (10 μM, 1% DMSO as a cosolvent) in the presence of various interferents.

Having established ER-P’s excellent fluorescence and PA response to polarity, we then examined the selectivity by the addition of various metal ions and amino acids in an aqueous solution of ER-P. Due to the high polarity of water, ER-P with or without these interfering substances displayed almost no fluorescence, whereas, the same concentration of ER-P exhibited high fluorescence in dioxane (Fig. 3A). Similarly, the PA intensity ratios of PA700/PA800 were almost unchanged in the presence of interferences, and a turn on response of the ratio emerged in the low polarity solvent dioxane (Fig. 3B). Further experiments demonstrated that ER-P was insensitive to pH values and viscosity (Fig. S2†), and ER-P showed excellent fluorescence and PA signal stability (Fig. S3 and S4†). Notably, compared with the commercial contrast agent indocyanine green (ICG), ER-P exhibited superior photostability and higher PA activity (Fig. S5 and S6†). In addition, an MTT assay suggested that ER-P has low cytotoxicity (Fig. S7†). In summary, ER-P is highly selective to polarity and has outstanding properties, which allow it to monitor polarity in certain media or even in live cells and *in vivo*.

### The subcellular localization of ER-P

**Fig. 4:**
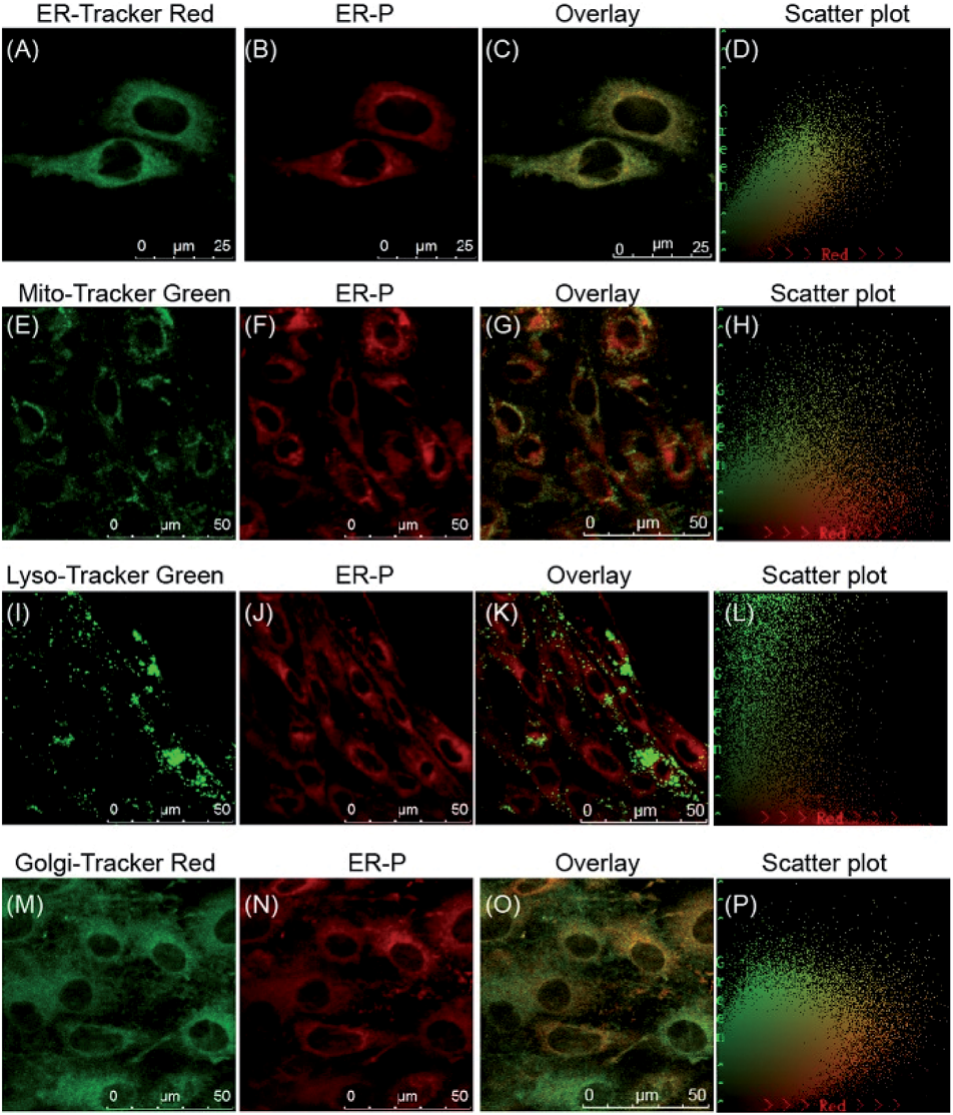
Confocal fluorescence images of HL-7702 cells showing co-staining of ER-P (10 μM, Ex = 633 nm, Em = 700–800 nm) with organelle dyes, including ER-Tracker Red (500 nM, Ex = 561 nm, Em = 580–630 nm), Mito-Tracker Green (100 nM, Ex = 488 nm, Em = 500–550 nm), Lyso-Tracker Green (100 nM, Ex = 488 nm, Em = 500–550 nm) and Golgi-Tracker-Red (500 nM, Ex = 561 nm, Em = 580–630 nm).

With this satisfactory outcome, we intended to further explore the subcellular distribution of ER-P by performing colocalization experiments in live cells. To begin with, HL-7702 cells were co-incubated with ER-P and various commercial organelle dyes, including ER-Tracker Red, Mito-Tracker Green, Lyso-Tracker Green and Golgi-Tracker Red. After the cells were washed with PBS, fluorescence images were captured by confocal microscopy. As expected, the overlapped fluorescence images in Fig. 4C indicated that the red fluorescence of ER-P in Fig. 4B overlaid well with the green fluorescence of the ER Tracker Red in Fig. 4A, with a colocalization coefficient of 0.91. This confirmed that the methyl sulphonamide moiety effectively guided ER-P into the ER.^48,49^ In contrast, ER-P displayed almost no fluorescence inside the mitochondria (Fig. 4E–H, colocalization coefficient 0.21) and lysosomes (Fig. 4I–L, colocalization coefficient 0.18). ER-P exhibited some fluorescence in the Golgi apparatus (Fig. 4M–P, colocalization coefficient 0.47), presumably due to the close physical contacts and entanglement between the ER and Golgi apparatus.^50^ The same experiment in HepG2 and HeLa cells also showed the prominent ER-targeting ability of ER-P (Fig. S8 and S9†). In addition, due to the negligible fluorescence in the culture medium, ER-P can still maintain a good signal to noise ratio without washing out the excess probe in comparison to the commercial ER Tracker Red (Fig. S10†), which will simplify the operations. In short, ER-P is a fascinating new probe with excellent biocompatibility and ER-targeting ability.

### Fluorescence visualization of polarity changes under ER stress

**Fig. 5:**
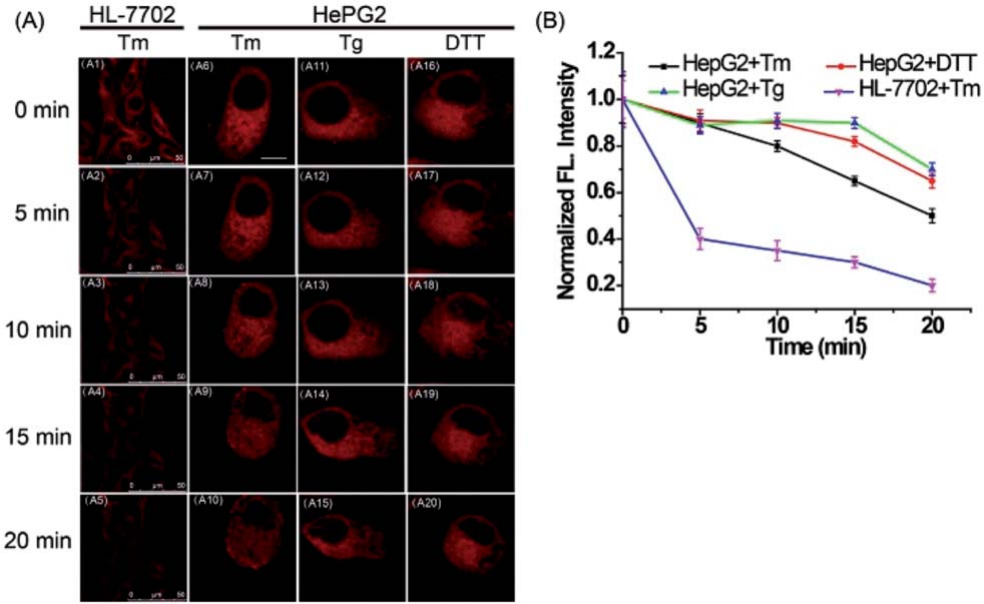
Fluorescence images of ER-P (10 μM) in live cells at different times after the addition of ER stress stimuli. (A1–A5) Fluorescence images of ER-P in HL-7702 cells after the addition of Tm (50 μg mL^–1^). Scale bar: 50 μm. (A6–A20) Fluorescence images of ER-P in HepG2 cells after the addition of Tm (50 μg mL^–1^), Tg (50 μg mL^–1^) or DTT (5.0 mM). Scale bar: 10 μm. (B) The average fluorescence intensity output of ER-P at different times. The excitation wavelength is 633 nm, and the collected emission wavelength ranges from 700 to 800 nm.

Next, to explore the fluorescence response of ER-P to ER polarity changes, the fluorescence intensity of ER-P in live cells under ER stress was detected. HL-7702 cells were incubated with ER-P. After being washed with PBS, the cells were treated with tunicamycin (Tm), which is produced by several bacteria and can inhibit glycosylation during protein or glycolipid synthesis in the ER, leading to acute ER stress.^51^ The fluorescence intensity of ER-P at different times is shown in Fig. 5A1–A5. The Tm-treated HL-7702 cells showed an obvious decrease in fluorescence intensity, suggesting an increase in polarity of the ER. Furthermore, the ER polarity of the HepG2 cells also increased gradually when stimulated with diverse ER stress stimuli (Fig. 5A6–A20), such as Tm, thapsigargin (Tg) and dithiothreitol (DTT). The increased polarity of the ER could be due to the substantial accumulation of polar proteins and the decline of nonpolar proteins in the ER under ER stress conditions.^52^ The previous results confirmed that cancer cells possessed lower mitochondrial polarity.^53,54^ To compare the ER polarity of normal and cancer cells, we applied ER-P to immunofluorescence staining using imaging flow cytometry within HepG2 and HL-7702 cells. The experimental data demonstrated that ER-P exhibited a higher fluorescence intensity in the HepG2 cells (Fig. S11†), predicting a lower polarity environment in the ER of the HepG2 cells. In brief, ER-P can be used to monitor the ER polarity variations under ER stress and distinguish ER polarity between normal and cancer cells by fluorescence imaging.

### Ratiometric PA imaging of polarity difference *in vivo*

**Fig. 6:**
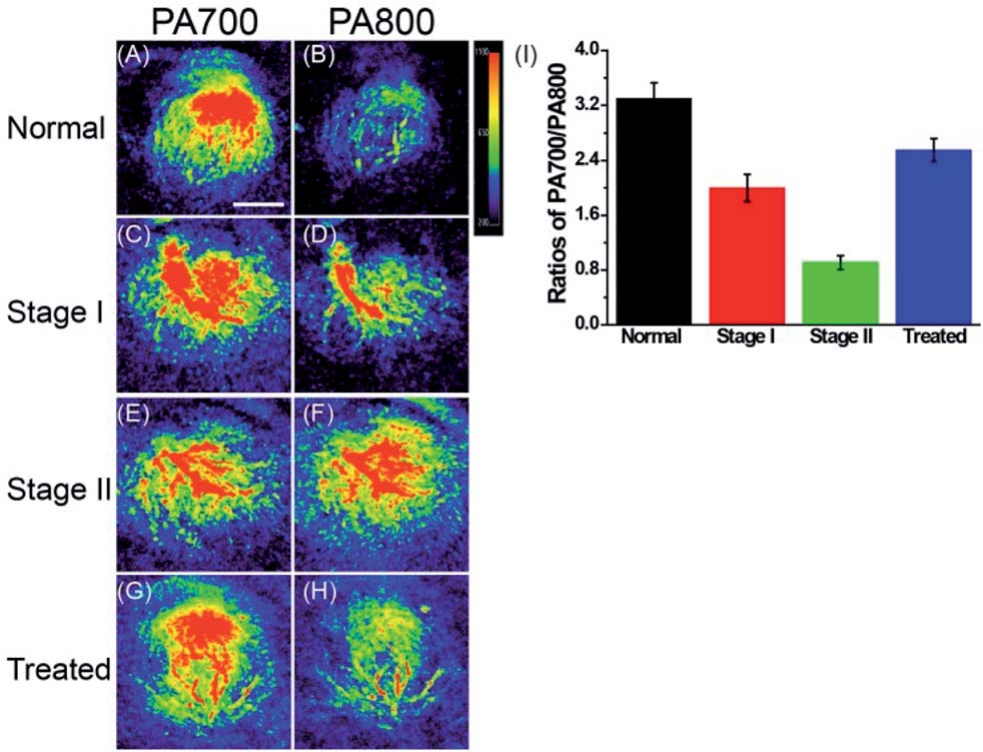
Photoacoustic images at two wavelengths of ER-P (100 μM) in the liver tissue of mice. (A), (C), (E) and (G) are photoacoustic images at 700 nm. (B), (D), (F) and (H) are photoacoustic images at 800 nm. Scale bar: 5.0 mm. (I) The average photoacoustic intensity output ratios between PA700 and PA800.

With this polarity-sensitive, excellent fluorescence and PA dual-mode probe in hand, we next studied whether ER-P could detect ER polarity differences in living animals and indicate diabetes-induced liver injury by a PA imaging technique. Kunming mice were purchased from the Shandong University Laboratory Animal Center. All of the animal experiments were approved and carried out in accordance with the relevant laws and guidelines issued by the Ethical Committee of Shandong University. ER-P was used to measure the polarity of liver tissue in normal, diabetic and drug-treated diabetic mice. Diabetic mice were obtained by treating normal mice with streptozotocin (STZ), an analog of *N*-acetylglucosamine that is a specific toxin for the pancreatic beta cell and can be used for preparing acutely diabetic animals.^55^ A portion of the diabetic mice were orally treated with metformin (Metf), which is a biguanide derivative used as an oral hypoglycaemic drug for diabetes, which has protective effects against liver injury in streptozotocin-diabetic rats.^1^ When diabetes was confirmed, ER-P was injected intraperitoneally to the normal, diabetic and Metf-treated diabetic mice. The mice at five days (stage I) and ten days (stage II) development of diabetes were selected to represent the different degrees of diabetes-induced liver injury. *In situ* PA imaging of the liver was performed on a PA system. As illustrated in Fig. 6, the PA700 intensity of ER-P in the liver tissue of normal mice was obviously higher than the PA800 intensity (Fig. 6A and B). In contrast, with enhanced liver injury induced by diabetes, the diabetic mice at stage I and stage II had higher and increasing PA800 intensities (Fig. 6C–F), and the PA800 intensity declined in the Metf-treated mice (Fig. 6G and H). We again calculated the PA intensity ratios PA700/PA800 (Fig. 6I), and the calculated data showed that the mean ratio in liver tissue of normal mice is 3.30, which indicates that the corresponding dielectric constant is 18.4 according to the linear relationship in Fig. 2B. However, lower mean ratios of 2.0 and 0.91 were present in the liver tissue of the diabetic mice at stage I and stage II, respectively, corresponding to dielectric constants of 38.4 and 55.2. After being treated with Metf, the mean ratio was 2.55, suggesting a dielectric constant of 29.9. Additionally, we also found that ER-P displayed decreased fluorescence intensity in the liver tissue of diabetic mice (Fig. S12†), further verifying the increase in polarity in the liver tissue. All of these data demonstrate that the liver tissue of diabetic mice possesses a higher polarity environment compared with normal mice, and after treatment with Metf, the polarity of the liver tissue recovers to some extent. Thus, ER-P is a prominent ratiometric PA imaging probe for imaging polarity differences in deep liver tissue, which can illustrate diabetic-induced liver injury.

## Conclusions

In conclusion, to nondestructively indicate diabetes-induced liver injury *in situ*, we have developed a fluorescence and PA imaging dual-mode probe, ER-P, for the detection of polarity. ER-P is highly sensitive and selective to environmental polarity and displays excellent fluorescence and PA properties. The fluorescence imaging results testify that ER-P can exclusively accumulate in the ER. Moreover, ER-P can monitor the increase in the ER polarity in live cells stimulated with ER stress stimuli. Remarkably, by utilizing a PA imaging technique, the ratiometric PA imaging results demonstrate that a higher polarity environment occurs in the liver tissue of diabetic mice, and after treatment with the antidiabetic drug metformin, the polarity of the liver tissue in diabetic mice decreased again. ER-P is proven to be a superior ratiometric PA imaging probe that can nondestructively detect the polarity of liver tissue in diabetic mice *in situ* and indicate the degree of diabetes-induced liver injury. Our present work may provide an ideal imaging technique for the early indication of diabetes-induced liver injury *in vivo*. Furthermore, this study will further prompt the application of PA imaging in the clinical diagnosis of diabetes-induced liver injury.

## Conflicts of interest

There are no conflicts to declare.
