# Supplementary material for: Ratiometric photoacoustic imaging of endoplasmic reticulum polarity in injured liver tissues of diabetic mice
Source: Chem Sci. 2017 Aug 14;8(10):7025–30. doi: 10.1039/c7sc02330h (PMC5642195; doi:10.1039/c7sc02330h)
Supplement: Supplementary file 1 [file SC-008-C7SC02330H-s001.pdf]

Electronic Supplementary Material (ESI) for Chemical Science.
This journal is © The Royal Society of Chemistry 2017

Ratiometric photoacoustic imaging of endoplasmic reticulum polarity in injured liver tissues of diabetic mice

Haibin Xiao, Chuanchen Wu, Ping Li*, Wen Gao, Wen Zhang, Wei Zhang, Lili Tong, and Bo Tang*

College of Chemistry, Chemical Engineering and Materials Science, Institute of Biomedical Sciences, Collaborative Innovation Center of Functionalized Probes for Chemical Imaging in Universities of Shandong, Key Laboratory of Molecular and Nano Probes, Ministry of Education, Shandong Normal University, Jinan 250014, PR China

E-mail: tangb@sdnu.edu.cn; lip@sdnu.edu.cn

Table of Contents

Materials and instruments

General procedure for fluorescence spectra detection

Measurement of relative fluorescence quantum yield

Cells/mice culture and imaging

Scheme S1 The facile synthesis of ER-P

Table S1 The detailed pectroscopic properties of ER-P in nine solvents

Figure S1 The fluorescence spectra of ER-P in dioxane-water mixtures

Figure S2 The fluorescence spectra of ER-P to pH values and viscosity

Figure S3 The fluorescence intensity stability of ER-P in different solvents

Figure S4 The PA signal stability of ER-P

Figure S5 The absorption photostability of ER-P and ICG

Figure S6 The PA signal activity of ER-P and ICG

Figure S7 The MTT assay of HepG2 cells with ER-P

Figure S8 The colocalization experiments of ER-P and organelle dyes in HepG2 cells

Figure S9 The colocalization experiments of ER-P and ER-Tracker Red in HeLa cells

Figure S10 The stain experiments of ER-P and ER tracker Red in HepG2 cells

Figure S11 The immunofluorescence staining of HL-7702 and HepG2 cells

Figure S12 The 3D fluorescence images in normal and diabetic mice

Materials and instruments

Unless otherwise stated, all reagents were purchased from commercial suppliers and used without further purification. The solvents were purified by conventional methods before use. ER-Tracker Red, Lyso-Tracker Green, Mito-Tracker Green and Golgi-Tracker Red were purchased from Invitrogen (USA). The 3-[4, 5-dimethylthiazol-2-yl]-2, 5 diphenyl tetrazolium bromide (MTT), indocyanine green (ICG), tunicamycin (Tm), thapsigargin (Tg), dithiothreitol (DTT) were purchased from Sigma Chemical Company. Silica gel (200-300 mesh) used for flash column chromatography was purchased from Qingdao Haiyang Chemical Co., Ltd. ER-P was dissolved in dimethyl sulfoxide (DMSO) to produce 1.0 mM stock solutions. ^{1}HNMR and ^{13}CNMR spectra were determined by 400 MHz and 100 MHz using Bruker NMR spectrometers. The mass spectra were obtained by Bruker maxis ultra-high resolution-TOF MS system. The fluorescence spectra measurements were performed using FLS-980 Edinburgh fluorescence spectrometer. Fluorescence imaging in cells were performed with Leica TCS SP8 Confocal Laser Scanning Microscope. The human cervical carcinoma HeLa cells, hepatocyte line HL-7702 and human hepatoma cells HepG2 were purchased from Cell Bank of the Chinese Academy of Sciences (Shanghai, China). The KM mice were purchased from Shandong University Laboratory Animal Center. All the animal experiments were carried out in accordance with the relevant laws and guidelines issued by the Ethical Committee of Shandong University.

General procedure for fluorescence spectra detection

ER-P was dissolved in dimethyl sulfoxide (DMSO) to produce 1.0 mM stock solution. The solution of the probe ER-P (10 μM) in different solvents or water-dioxane mixtures was obtained by diluting the probe stock solution with corresponding media. The fluorescence spectra were recorded immediately after appropriate testing species were mixed with the probe. Unless otherwise noted, for all the measurements, the excitation wavelength is 633 nm, and the excitation/emission slit widths are 4/4 nm.

Measurement of relative fluorescence quantum yield[1,2]

Fluorescence quantum yield was determined by using ICG (Φ_f=0.13 in DMSO) for ER-P as a fluorescence standard. The quantum yield was calculated using the following equation:

$\Phi_x=\Phi_s(A_sF_x/A_xF_s)(n_x/n_s)^2$

Where Φ_x is the fluorescence quantum yield, A is the absorbance at the excitation wavelength, F is the area under the corrected emission curve, and n is the refractive index of the solvents used. Subscripts S and X refer to the standard and to the unknown, respectively. For ER-P and ICG, the excitation wavelength was at 650 nm while keeping the absorption below 0.05.

Cells/mice culture and imaging

Cells were cultured in high glucose DMEM (4.5 g of glucose/L) supplemented with 10% fetal bovine serum, 1% penicillin, and 1% streptomycin at 37 °C in a 5% CO2 /95% air incubator MCO-15AC (SANYO, Tokyo, Japan). One day before imaging,

the cells were detached and were replanted on glass-bottomed dishes.

The mice were intraperitoneally injected with STZ freshly dissolved in 0.01 mol/L citrate buffer (pH 4.5) at a dose of 150 mg/kg body weight (BW) after overnight fasting. The diabetes of mouse was confirmed by the presence of hyperglycemia (blood glucose level ≥ 16.7 mmol/L) 72 h after STZ injection. Then a portion of the diabetic mice were orally treated with PBS or Metf, respectively. After 10 days, ER-P was injected intraperitoneally to normal, diabetic and Metf-treated diabetic mice. In situ PA imaging of the liver tissue was performed on a PA system.

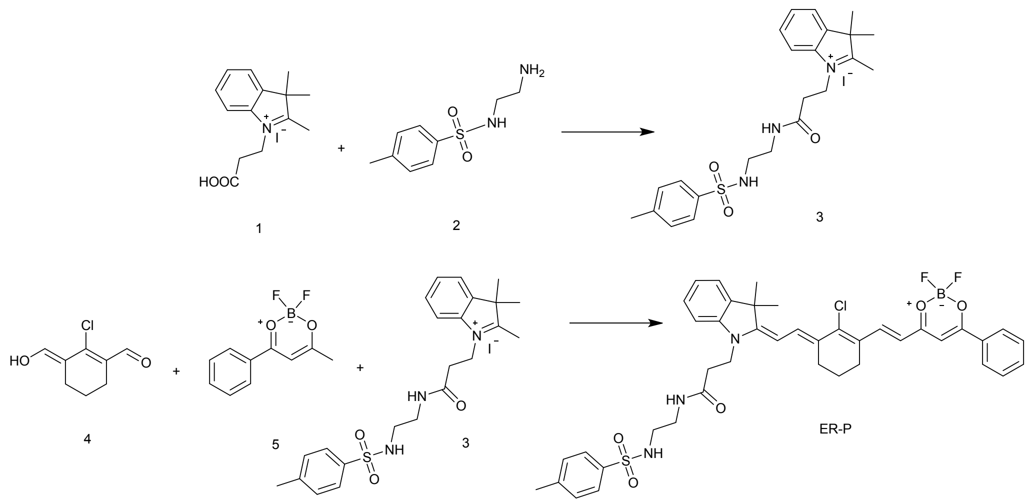

Scheme S1. The facile synthesis of **ER-P**

The compounds **1**, **2**, **4**, **5** were synthesized following by the previous reports.[3-6]

Compound 3: Compound **1** (0.359 g, 1.0 mmol) was dissolved in dimethylformamide (4.0 mL) and dichloromethane (8.0 mL) together with triethylamine (1.0 mL), 1-hydroxybenzotriazole (0.212 g, 1.0 mmol) and 1-(3-dimethylaminopropyl)-3-ethylcarbodiimide hydrochloride (0.192 g, 1.0 mmol). The mixture was stirred at 0 °C for 30 min, then compound **2** (0.214 g, 1.0 mmol) was added. The mixture was reacted at 25 °C for 24 h. Compound **3** was precipitated by addition of 300 mL diethyl ether into the concentrated liquid. And it was used to next step without further purification.

Probe ER-P: Under Ar gas atmosphere, compound **4** (0.172 g, 1.0 mmol), **5** (0.21 g, 1.0 mmol) and sodium acetate were dissolved in 15 mL acetic anhydride. The mixture was stirred at 65 °C for 4 h, then compound **3** (0.55 g, 1.0 mmol) was added. After reacted at 65 °C for another 4 h, the mixture was poured into 100 mL of saturated $NaHCO_3$ solution and mixed carefully. The solid was collected with dichloromethane after the aqueous solution was poured off. The organic layers were dried over Na_2SO_4, and evaporated under reduced pressure. Probe ER-P (0.19 g, yield 25%) was obtained as green solid by column chromatography on silica gel flash chromatography using CH_2Cl_2. ^{1}H NMR (400 MHz, *d6*-DMSO): 1.571 (s, 6H), 1.803 (t, J=5.2 Hz, 2H), 2.381 (s, 3H), 2.431 (t, J=6.4 Hz, 2H), 2.577-2.624 (m, 6H), 3.005 (q, J=6.4 Hz, 2H), 4.132 (t, J=5.2 Hz, 2H), 6.444 (d, J=14.4 Hz,1H), 5.93 (d,J=13.2 Hz,1H),7.112 (s, 1H), 7.000-7.064 (m, 2H), 7.238 (t, J=7.6 Hz, 1H), 7.389 (t, J=8.4 Hz, 4H), 7.562 (t, J=6.8 Hz, 1H), 7.617 (d, J=8.0 Hz, 4H), 7.699 (t, J=7.6 Hz, 1H), 7.883 (d,J=13.2 Hz,1H),

8.079 (d, J=8.0 Hz, 2H), 8.354 (d, J=14.4 Hz,1H). ^{13}C NMR (100 MHz, *d6*-DMSO): 21.10, 21.49, 26.14, 27.09, 28.33, 33.20, 39.14, 42.16, 47.27, 96.33, 98.73, 108.93, 116.62, 122.13, 122.36, 125.28, 126.88, 127.12, 128.18, 128.27, 129.39, 129.82, 132.77, 134.16, 135.77, 137.90, 139.72, 142.84, 143.24, 144.24, 145.92, 164.71, 170.39, 176.17, 179.05, 183.63. HRMS (ESI) m/z calcd. for $C_{41}H_{43}BF_2ClN_3O_5S$ [M+Na$^+$]: 796.2572, found 796.2658.

Table S1. The detailed pectroscopic properties of ER-P in seven solvents.

Solvent	ε[a]	P[b]	λ_{abs}/nm	λ_{em}/nm	Stokes shift/nm	ε[c]/M^{-1} cm^{-1}	$Ø_f$[d]
DMSO	47.2	7.2	794	820	26	74700	0.89%
Ethylene glycol	37.7	6.9	770	816	46	66900	1.1%
Acetonitrile	37.5	6.2	750	816	66	65200	1.5%
Acetone	20.7	5.4	726	812	86	66000	5.0%
Chloroform	4.80	4.4	708	806	98	68400	6.0%
Tetrahydrofuran	7.50	4.2	706	802	96	54100	10.8%
Toluene	2.37	2.4	694	786	92	63600	12.4%

Superscript a, b, c and d represent the dielectric constant of solvents, polarity index of solvents, molar extinction coefficient, and relative fluorescence quantum yield, respectively.

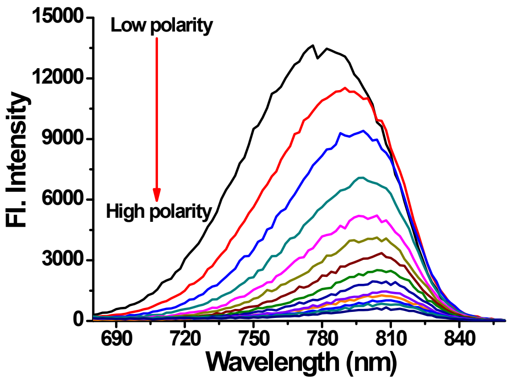

Figure S1. The fluorescence spectra of ER-P (10 μM, 1% DMSO as a cosolvent) in dioxane-water mixture solvents. The excitation wavelength was 633 nm.

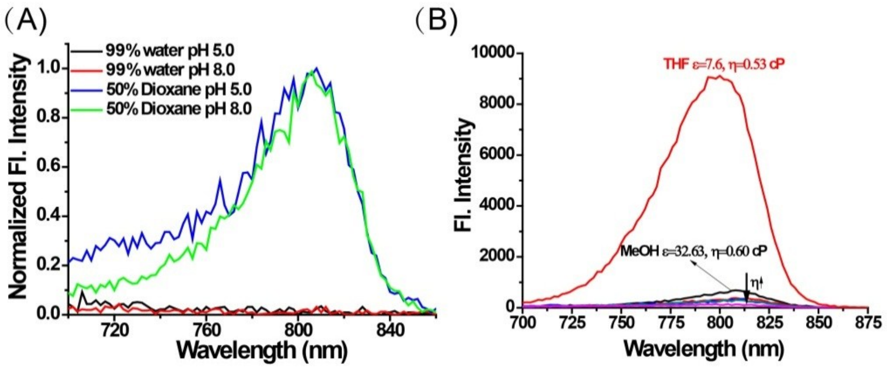

Figure S2. The fluorescence spectra of ER-P (10 μM, 1% DMSO as a cosolvent) to pH values and viscosity. (A) The fluorescence spectra of ER-P under different pH values buffer. The under two lines contain 99% water with corresponding pH values, and the green and blue lines contain 50% water under pH 5.0 and 8.0. (B) The fluorescence spectra of ER-P in methanol-glycerol system under different viscosity. THF and methanol have almost the same viscosity (0.53 cP *vs* 0.60 cP) but different polarity (ε=7.6 *vs* 32.63).The fluorescence intensity of ER-P displayed huge difference in them. The fluorescence intensity changed little with increasing viscosity from 0.60 cP to about 100 cP. The excitation wavelength was 633 nm.

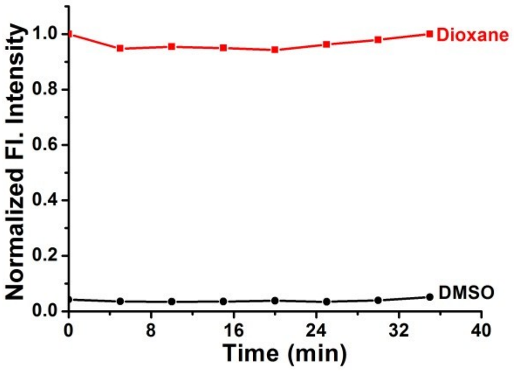

Figure S3. The fluorescence intensity stability of ER-P (10 μM) in different solvents.

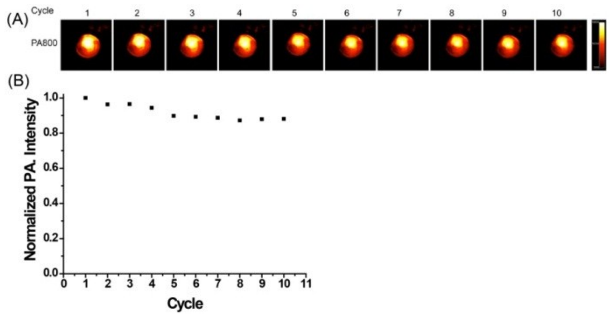

Figure S4. (A) The PA intensity images of ER-P (10 μM) upon excited with 800 nm under continuous ten cycles. (B) The output of average PA intensity of ER-P from images A. It's obvious that ER-P still keep excellent PA intensity after continuous ten

cycles.

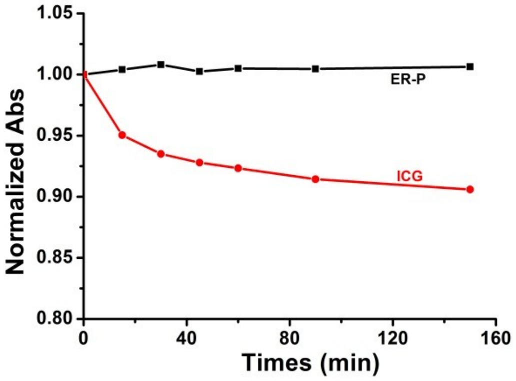

Figure S5. The absorption photostability of ER-P (10 μM) and ICG (10 μM) under exposure to sunlight. The absorption of ER-P was almost changeless during 150 min, however, the absorption of ICG decline to some extent.

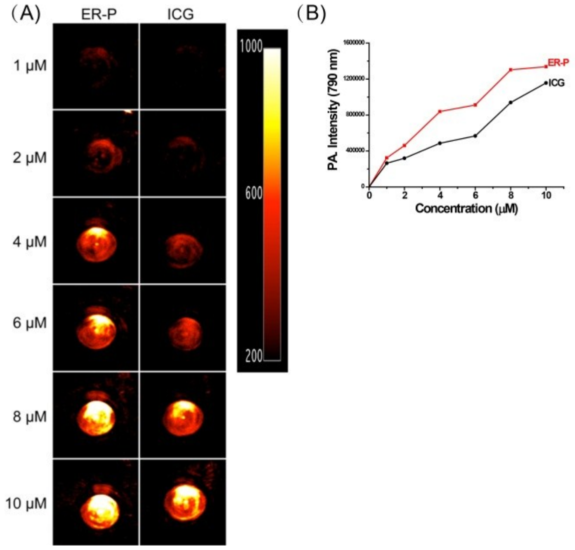

Figure S6. The PA signal activity of ER-P (10 μM) and ICG (10 μM). (A) The PA intensity images of ER-P and ICG at the same concentration upon excited with 790 nm. (B) The output of PA intensity of ER-P and ICG with concentration from images A. It's obvious that ER-P possesses higher PA activity compared with ICG.

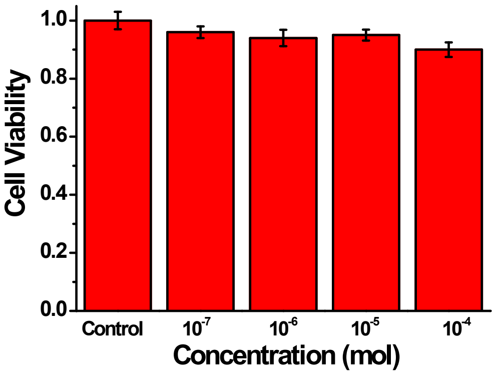

Figure S7. The MTT assay of HepG2 cells with different concentrations of ER-P. The IC_{50} value was calculated to be 324 μM.

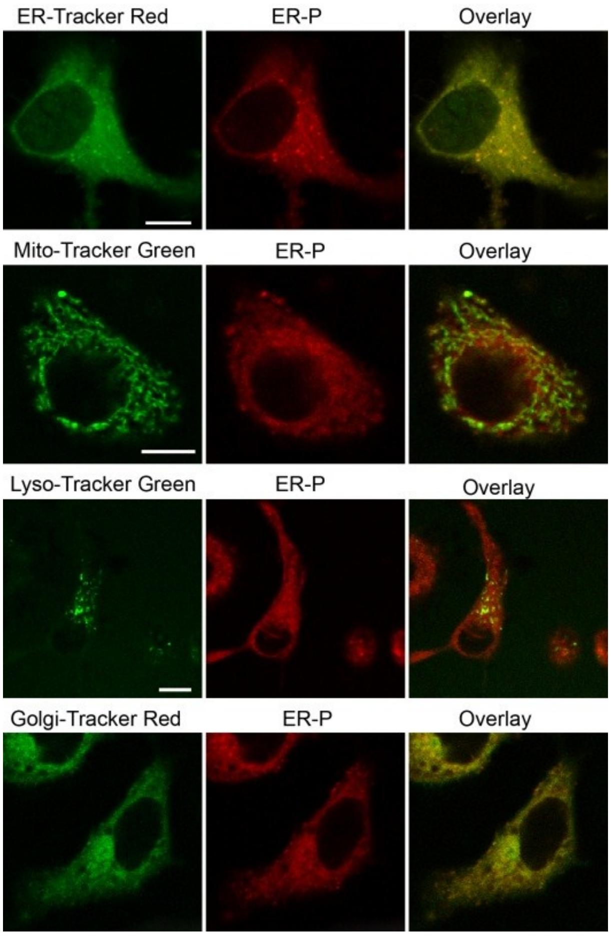

Figure S8. The confocal fluorescence images of HepG2 cells showing co-staining of ER-P (10 μM, Ex=633 nm, Em=700-800 nm) with various organelle dyes, including ER-Tracker Red (500 nM, Ex=561 nm, Em=580-630 nm), Mito-Tracker Green (100 nM, Ex=488 nm, Em=500-550 nm), Lyso-Tracker Green (100 nM, Ex=488 nm, Em=500-550 nm) and Golgi-Tracker-Red (500 nM, Ex=561 nm, Em=580-630 nm). The colocalization coefficient is 0.92, 0.19, 0.17, 0.53 for ER, Mito, Lyso, and Golgi apparatus respectively. Scale bar: 10 μm.

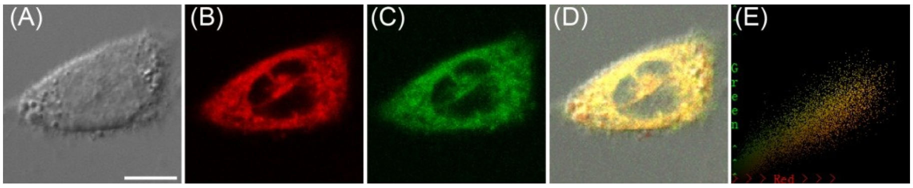

Figure S9. The confocal fluorescence images of HeLa cells showing co-staining of ER-P (10 μM, Ex=633 nm, Em=700-800 nm) with ER-Tracker Red (500 nM, Ex=561 nm, Em=580-630 nm). The colocalization coefficient is 0.90. Scale bar: 10 μm.

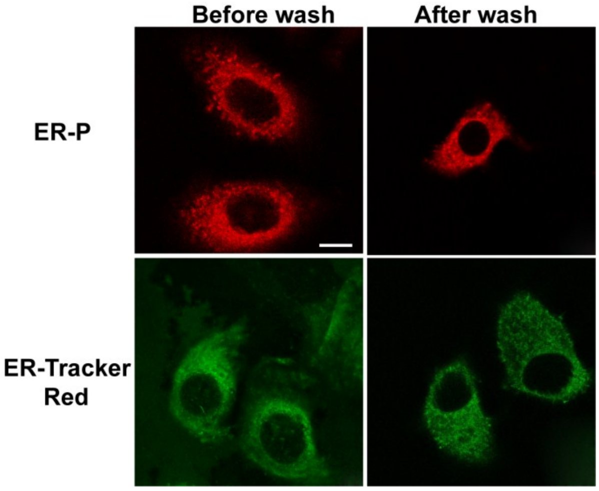

Figure S10. The stain experiments of ER-P (10 μM) and ER tracker Red (500 nM) in HepG2 cells. In the condition of washing or no-washing, ER-P both keep good signal noise ratio. In sharp contrast, ER-Tracker Red displayed intense background fluorescence before washing out the excess dye. Scale bar: 10 μm.

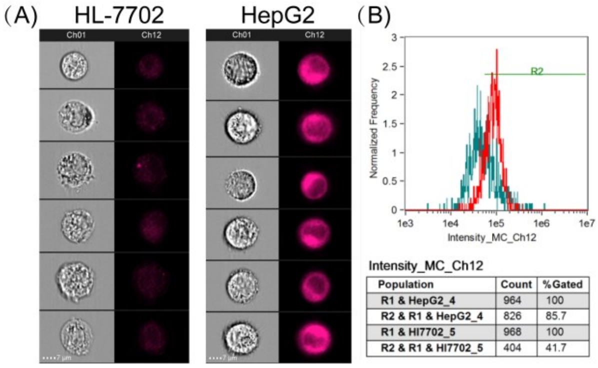

Figure S11. The immunofluorescence staining of HL-7702 and HepG2 cells using imaging flow cytometry. (A) Cell images after incubated with ER-P (10 μM, Ex=642 nm, Em=745-800 nm). Scale bar: 7.0 μm. (B) Flow cytometry data for the fluorescence intensity of two kinds of cells under corresponding to the cells in (A). This data demonstrated that ER-P exhibited higher fluorescence intensity in HepG2 cells, indicatinging lower polarity environment in ER of HepG2 cells.

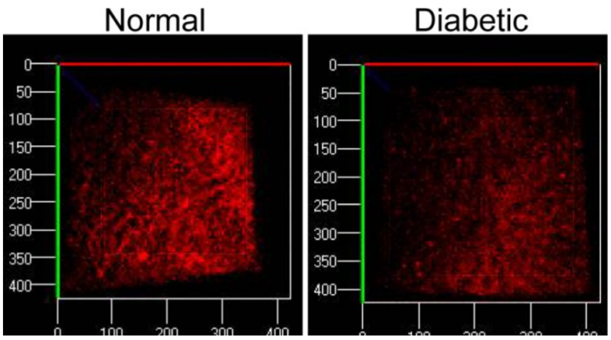

Figure S12. The 3D fluorescence imaging of polarity difference in liver tissues of normal and diabetic mouse. After anaesthetized, the liver of the mice was surgically exposed and injected with ER-P (100 μM, 50 μL, 10% DMSO). The fluorescence images were collected from 700-800 nm upon excited with 633 nm laser. ER-P displayed decreased fluorescence intensity in liver tissue of diabetic mice, suggesting the rise of polarity in liver tissue.

References

(1) D. Magde, G. E. Rojas and P. G. Seybold, *Photochem. Photobiol.*, 1999, **70**, 737-744.

(2) D. Oushiki, H. Kojima, T. Terai, M. MArita, K. Hanaoka, Y. Urano and T. Nagano, *J. Am. Chem. Soc.*, 2010, **132**, 2795-2801.

(3) Z. Ma, Y. Lin, Y. Cheng, W. Wu, R. Cai, S. Chen, B. Shi, B. Han, X. Shi, Y. Zhou, L. Du and M. Li, *J. Med. Chem.*, 2016, **59**, 2151-2162.

(4) M. T. Barros and F. Siñeriz, *Tetrahedron* 2000, **56**, 4759-4764

(5) F. Kong, R. Liu, R. Chu, X. Wang, K. Xu and B. Tang, *Chem. Commun.*, 2013, **49**, 9176-9178.

(6) H. D. Ilge, E. Birckner, D. Fassler, M. V. Kozmenko, M. G. Kuz'min and H. Hartmann, *Journal of photochemistry* 1986, **32**, 177-189.
